# Priming Cross-Protective Bovine Viral Diarrhea Virus-Specific Immunity Using Live-Vectored Mosaic Antigens

**DOI:** 10.1371/journal.pone.0170425

**Published:** 2017-01-18

**Authors:** Shehnaz Lokhandwala, Xin Fang, Suryakant D. Waghela, Jocelyn Bray, Leo M. Njongmeta, Andy Herring, Karim W. Abdelsalam, Christopher Chase, Waithaka Mwangi

**Affiliations:** 1 Department of Veterinary Pathobiology, Texas A&M University, College Station, Texas, United States of America; 2 Department of Animal Science, Texas A&M University, College Station, Texas, United States of America; 3 Department of Veterinary and Biomedical Sciences, South Dakota State University, Brookings, South Dakota, United States of America; Instituto Butantan, BRAZIL

## Abstract

Bovine viral diarrhea virus (BVDV) plays a key role in bovine respiratory disease complex, which can lead to pneumonia, diarrhea and death of calves. Current vaccines are not very effective due, in part, to immunosuppressive traits and failure to induce broad protection. There are diverse BVDV strains and thus, current vaccines contain representative genotype 1 and 2 viruses (BVDV-1 & 2) to broaden coverage. BVDV modified live virus (MLV) vaccines are superior to killed virus vaccines, but they are susceptible to neutralization and complement-mediated destruction triggered by passively acquired antibodies, thus limiting their efficacy. We generated three novel mosaic polypeptide chimeras, designated N^pro^E2^123^; NS^231^; and NS^232^, which incorporate protective determinants that are highly conserved among BVDV-1a, 1b, and BVDV-2 genotypes. In addition, strain-specific protective antigens from disparate BVDV strains were included to broaden coverage. We confirmed that adenovirus constructs expressing these antigens were strongly recognized by monoclonal antibodies, polyclonal sera, and IFN-γ-secreting T cells generated against diverse BVDV strains. In a proof-of-concept efficacy study, the multi-antigen proto-type vaccine induced higher, but not significantly different, IFN-γ spot forming cells and T-cell proliferation compared to a commercial MLV vaccine. In regards to the humoral response, the prototype vaccine induced higher BVDV-1 specific neutralizing antibody titers, whereas the MLV vaccine induced higher BVDV-2 specific neutralizing antibody titers. Following BVDV type 2a (1373) challenge, calves immunized with the proto-type or the MLV vaccine had lower clinical scores compared to naïve controls. These results support the hypothesis that a broadly protective subunit vaccine can be generated using mosaic polypeptides that incorporate rationally selected and validated protective determinants from diverse BVDV strains. Furthermore, regarding biosafety of using a live vector in cattle, we showed that recombinant human adenovirus-5 was cleared within one week following intradermal inoculation.

## Introduction

Bovine viral diarrhea virus (BVDV), an infectious pathogen that is prevalent in cattle herds globally, is a key agent responsible for causing Bovine Respiratory Disease Complex (BRDC) [1]. Infection with BVDV can cause severe diarrhea, respiratory disease, immunosuppression, abortion, congenital malformations, and birth of persistently infected (PI) calves, which play a major role in virus transmission in herds [2]. Immunosuppression caused by acute infection of unprotected calves allows secondary infections to establish and cause pneumonia or enteritis [3]. The secondary infections are responsible for high rates of morbidity and mortality, and it is estimated that the U.S. livestock industry loses >$1billion annually due to BRDC [4, 5].

This virus is classified as a member of the genus Pestivirus within the family *Flaviviridae* [6]. Two BVDV genotypes (type 1 and 2) are recognized according to serological and genetic relatedness [7]. The BVDV isolates circulating in the world are heterogeneous: BVDV genotype 1 (BVDV-1) is subdivided into a minimum of 12 sub-genotypes (BVDV1a, b, c.…l), whereas BVDV genotype 2 (BVDV-2) is classified into 4 subtypes, 2a-2d [8, 9]. The BVDV can also be divided into cytopathic and non-cytopathic biotypes (cpBVDV and ncpBVDV, respectively), based on their lytic effects on infected cells. The BVDV isolates cause a wide range of disease manifestations, which include sub-clinical and persistent infections, fetal infections, and host immunosuppression [10]. Infected cattle begin to shed the virus into the environment for about ten continuous days starting as early as four days after subclinical infection, whereas PI animals shed the virus for their entire lifetime [11, 12]. The prevalence of PI animals in selected herds in the United States is estimated at 1.7% of the cattle population, and these animals are considered to be the primary source of infection of susceptible animals [13].

BVDV infection in cattle induces high titers of neutralizing antibodies that prevent reinfections especially with the same genotype/sub-genotype [14, 15]. Some studies have demonstrated prevention of clinical signs, but not viral shedding, in cattle upon challenge with BVDV-2 following immunization with BVDV-1 [16, 17]. Failure of vaccination has been attributed to infection with variant genotype(s) as well as development of antigenically distinct viruses in exposed animals [18, 19]. Individual PI cattle may also be a source of genetic variants that amplify following infection of susceptible cattle [20, 21]. However, in the absence of neutralizing antibodies, mutations occur faster and more frequently in BVDV following infection of pregnant animals [22]. Many of the virus genome mutations result in amino acid changes in E2 glycoprotein, a key target of the neutralizing antibodies [21, 23]. The E2 glycoprotein is highly immunogenic and at least nine epitopes have been mapped within three antigenic domains [24–28]. One of these antigenic determinants is immunodominant in BVDV-1 and there are three in BVDV-2 that induce neutralizing antibodies in animals [25]. However, it is also reported that viremia can occur despite the presence of neutralizing antibodies in infected animals, and some animals can be protected against BVDV infection in the absence of E2-specific neutralizing antibodies, suggesting a role for neutralizing epitopes from other antigens and/or T cells in protection [29, 30]. Clearance of BVDV infections has also been associated with strain-specific MHC-restricted CD4^+^ and CD8^+^ T-cell responses [15, 31]. Cell mediated response to infection is initially provided by E2 and NS2-3 antigen-specific helper CD4^+^ T-cells [32–34].

Despite availability of vaccines, BVDV prevalence has not markedly reduced due, in part, to failure of the vaccines to confer broad protection [35, 36]. Currently, both killed and modified live virus (MLV) vaccines are commercially available [37]. The killed vaccine elicits primarily a humoral response with minimal cell mediated response, whereas MLV vaccines are better at inducing CD4^+^ and CD8^+^ T-cells responses in addition to antibody responses [38]. Since the presence of BVDV-specific maternal antibodies interferes with efficacy of BVDV vaccines, especially MLV, immunization is usually delayed until most of the maternal BVDV antibodies have waned [19, 39]. However, BVDV-specific antibodies in each animal decline at different rates and thus, antibody titers in some calves fall below protective levels much earlier than expected, and in the presence of PI calves in the herd, there is a high risk of infection [40]. MLV vaccines are currently the most efficacious, but genotype-specific vaccines are not effective at conferring cross-protection and thus, protection against BVDV-1 and 2 requires a vaccine formulation that contains a representative of genotype type 1 and 2 viruses. The MLV vaccines are not considered to be safe since the attenuated virus can revert to wild type virus, cause in-utero infections and mucosal disease, carry the risk of vaccine contamination with adventitious viruses, and are immunosuppressive [41, 42]. Furthermore, MLV strains may cause ovarian lesions leading to infertility in cows [43]. Both killed and MLV vaccine virus are traditionally grown in MDBK cells and recent findings show that calves fed colostrum from some dams vaccinated with killed BVDV vaccine formulated with adjuvant have a high incidence of a syndrome characterized by spontaneous bleeding, severe anemia with heavy bone marrow damage. There is evidence to show that the damage is due to maternal alloantibodies induced by the vaccines against bovine cell antigens, including MHC-I molecules, and the syndrome has been named bovine neonatal pancytopenia [44–46].

Given the limitations of the current vaccines, there is a need to develop improved vaccines for safe, robust, and broad protection against diverse BVDV genotypes. Empirical selection and validation of protective immune targets that are conserved among diverse BVDV strains can be used to generate novel mosaic antigens for development of a contemporary vaccine. Similar strategies have been used to develop broadly protective vaccines to overcome a wide Influenza and HIV-1 genetic diversity [47–49]. The BVDV envelop (E2) and non-structural (NS2-3) antigens are immunodominant, and neutralizing antibody as well as T-cell responses directed against these antigens can confer protection [50, 51]. Importantly, evaluation of BVDV-specific immune responses following resolution of acute infection has revealed that the E2, NS2-3, and N-terminal protease fragment (N^pro^) antigens contain CD4^+^ T-cell epitopes [31]. In addition, MHC *DR*-restricted T-cell epitopes have been identified from conserved regions of E2 and NS2-3 [31, 34, 51–53].

In this study, we generated a prototype vaccine composed of recombinant adenoviruses expressing three novel mosaic polypeptide chimeras, designated N^pro^E2^123^; NS^231^; and NS^232^. These antigens incorporated neutralizing epitopes, defined and predicted IFN-γ-inducing CD4^+^ T-cell as well as cytotoxic T lymphocyte determinants that are highly conserved among BVDV-1a, b, and BVDV-2 genotypes [24, 25, 31, 51, 52]. In addition, strain-specific protective antigens from disparate BVDV strains whose genome sequences are available were included to broaden coverage. We compared the immunogenicity and protective efficacy of this prototype adenovirus-vectored vaccine to a commercial MLV vaccine in calves.

Adenovirus-vectored subunit vaccines are undergoing clinical trials in readiness for deployment [54, 55]; there is concern that persistence of the construct in host tissues may increase chances of generating replication-competent progenies if recombination with closely related viruses occurs. Thus, we set out to determine replication-incompetent recombinant human adenovirus-5 persistence at the skin injection site, the draining lymph node, and the spleen of calves following intradermal inoculation.

## Materials and Methods

### Design of genes encoding novel mosaic BVDV antigens

Published reports on protective BVDV antibody and T-cell epitopes, sequenced genomes, and bioinformatics tools were used to design novel mosaic polypeptides, which incorporated consensus and strain-specific key antigenic determinants from BVDV-1 and 2 strains [24, 25, 31, 47, 48, 52, 56, 57]. Analysis of sequenced genomes showed that the N^pro^ antigen is highly conserved, but the E2 and the NS2-3 antigens have conserved and variable domains. Amino acid sequences of the E2 proteins from currently defined BVDV-1 or BVDV-2 genotypes were aligned and three novel mosaic E2 polypeptides, designated E2^1,2,3^ (E2^1-3^), each containing consensus E2 determinants plus defined strain-specific neutralization epitopes were selected, and wherever there was no consensus at a specific amino acid position for the BVDV-1 genotypes, amino acid from the BVDV-1b sequence was selected since this is the most prevalent sub-type in North America. The E2^1-3^ polypeptide sequence was fused in-frame to the C-termini of the N^pro^ polypeptide and the resultant chimeric polypeptide, designated N^pro^E2^1-3^, was used to generate a codon-optimized synthetic gene, designated *n*_*pro*_-*e2*_*1a*_*-e2*_*1b*_*-e2*_*2*_ (*n*_*pro*_*e2*_*1-3*_,), that also included *flag* tag sequence at the 3’ end. Two additional mosaic polypeptides that incorporated consensus amino acids from diverse NS2-3 proteins, designated NS^2-31^ (from BVDV-1 genotypes) and NS^2-32^ (from BVDV-2 genotypes) were similarly designed and used to generate two synthetic gene sequences, designated *ns2-3*_*1*,_ and *ns2-3*_*2*_, respectively, that also included the *flag* tag sequence at the 3’ end. Synthetic genes were codon-optimized, custom-made, cloned into pUC57 vector, and sequence-verified by GenScript Inc., NJ, USA.

### Generation of recombinant adenovirus plasmid expression constructs

The three synthetic genes (*n*_*pro*_*e2*_*1-3*_, *ns2-3*_*1*,_ and *ns2-3*_*2*_) were subcloned into pDonR vector using the Gateway Technology (Life Technologies, NY, USA) to generate shuttle constructs. Positive clones were identified by PCR screening of plasmid DNA in bacteria colonies using vector-specific forward primer and gene-specific reverse primer. Authentic entry constructs, designated pDonRN^pro^E2^1-3^, pDonRNS^2-31^, and pDonRNS^2-32^, respectively were selected by DNA sequencing. The selected constructs were used to transfer each gene into pAd adenovirus plasmid backbone by homologous recombination (Gateway Technology, Life Technologies, NY, USA) and recombinant constructs were identified by PCR screening as above. Authentic recombinant plasmid constructs, designated pAdN^pro^E2^1-3^, pAdNS^2-31^, and pAdNS^2-32^, respectively were selected after DNA sequencing.

### Protein expression by plasmid constructs and generation of recombinant adenoviruses

Protein expression was evaluated by immunocytometric analysis of human embryonic kidney (HEK)-293A cells grown in 12-well tissue culture plates and transfected with 1 μg of the selected clones of the pAd DNA constructs, and then probed with anti-FLAG mAb at 48 hr. post-transfection as previously described [58]. Five clones of each pAd construct were selected based on efficiency of protein expression as judged by the immunocytometric analysis, and 2 μg DNA of each construct was digested with Pac-I restriction enzyme. The digested DNA was transfected into HEK-293A cells grown in 6-well plates to generate recombinant adenoviruses that were designated AdN^pro^E2^1-3^, AdNS^2-31^, and AdNS^2-32^, respectively. In addition, adenovirus expressing luciferase (AdLuc) was generated to serve as a negative control. Protein expression by the AdN^pro^E2^1-3^, AdNS^2-31^, and AdNS^2-32^ adenoviruses was tested and validated by immunocytometric analysis of infected HEK-293A cells as above, whereas fluorescence was used to confirm luciferase expression.

One clone of each recombinant adenovirus was selected for amplification based on protein expression. The bulk viruses were tested for protein expression as above and following titer determination, replication competence of the recombinant adenoviruses was determined by immunocytometric analysis of HEK-293A (which supports adenovirus replication) and MDBK cells (susceptible to adenovirus infection, but do not support replication of replication-incompetent adenovirus) infected overnight with one MOI of each virus construct and then probed with an in-house generated rabbit anti-adenovirus polyclonal IgG (1:500 dilution) followed by an alkaline-phosphatase-conjugated anti-Rabbit IgG (1:1000) (Jackson ImmunoResearch, Cat #711-055-152) secondary antibody and Fast Red TR–Naphthol AS-MX as the substrate (Sigma, F4523) to evaluate infectivity.

### Validation of the mosaic antigens using BVDV-specific antibodies and T-cells

Authenticity of the mosaic N^pro^E2^1-3^, NS^2-31^, and NS^2-32^ antigens was confirmed by immunocytometric analysis using E2-specific neutralizing monoclonal antibodies (mAbs) and polyclonal antibodies (pAbs) generated against diverse BVDV strains. Briefly, HEK-293A cells grown in 12-well plates were infected for 48 hr. with AdN^pro^E2^1-3^, AdNS^2-31^, AdNS^2-32^, or AdLuc and probed with anti-BVDV E2 mAbs 348 and 26A (VMRD, Inc., Pullman, WA), goat anti-BVDV polyclonal sera (VMRD), and bovine anti-BVDV hyperimmune sera from steers immunized and challenged with multiple BVDV-1 and 2 strains [59]. Antigen authenticity was further confirmed by ELISA and Western Blot analysis using the above mentioned antibodies.

The authenticity of the T-cell epitopes in the mosaic antigens was validated by proliferation assays using peripheral blood mononuclear cells (PBMCs) isolated from the BVDV-immunized steers [59]. Recombinant N^pro^E2^1-3^, NS^2-31^ and NS^2-32^ antigens were expressed by using recombinant baculoviruses in High Five cells (Thermo Fisher Scientific) generated using the Bac-to-Bac HBM TOPO Secreted Expression System (Thermo Fisher Scientific) as per manufacturer’s instructions and validated as above. These antigens were then affinity purified using Anti-FLAG M2 Affinity Gel (Sigma) and used at 5μg/ml to conduct ^3^H-Thymidine incorporation assays to quantify antigen-specific T cell responses as previously described [58]. Heat killed BVDV-1b (CA0401186a) and BVDV-2 (A125) at 5μg/ml served as positive control antigens, whereas medium alone was the negative control. The outcome of the cell proliferation was presented as counts per minute (cpm).

### Immunization and challenge of calves

Three groups (A, B, and C), of age-matched BVDV sero-negative and virus-free weaned Holstein calves (n = 5) were identified as previously described [60] and used in this study as shown in Table 1. Each calf in group A was inoculated subcutaneously (SQ) with a cocktail, designated AdBVDV, containing the AdN^pro^E2^1-3^, AdNS^2-31^, and AdNS^2-32^ recombinant adenoviruses (5 x 10^10^ TCID_50_/construct) formulated in adjuvant E (BenchMark-Vaxliant). Each calf in group B was similarly inoculated, but with a commercial MLV BVDV-1 and 2 vaccine (Bovi-Shield Gold^™^, Zoetis Inc., Kalamazoo, MI), whereas each calf in group C was inoculated with the recombinant AdLuc formulated in adjuvant E. Seventy-nine days post-priming, the AdBVDV vaccinees and the negative controls received inoculation of the respective priming immunogen and dose as above. One hundred and forty nine days post-boosting, all the calves were challenged by intranasal administration of 2 x 10^6^ TCID_50_ of BVDV-1373 using a human nasal atomizer. (http://www.teleflexarcatalog.com/anesthesia-respiratory/airway/categories/552).

**Table 1:** Immunization Protocol.

Calf ID	Vaccine-Prime	Vaccine-Boost
4	AdBVDV	AdBVDV
12	AdBVDV	AdBVDV
13	AdBVDV	AdBVDV
22	AdBVDV	AdBVDV
23	AdBVDV	AdBVDV
3	BVDV MLV	-
14	BVDV MLV	-
19	BVDV MLV	-
24	BVDV MLV	-
27	BVDV MLV	-
10	AdLuciferase	AdLuciferase
18	AdLuciferase	AdLuciferase
25	AdLuciferase	AdLuciferase
28	AdLuciferase	AdLuciferase
29	AdLuciferase	AdLuciferase

Calves in the treatment group were inoculated subcutaneously with a cocktail of the AdN^pro^E2^1-3^, AdNS^2-31^, and AdNS^2-32^ recombinant adenoviruses (AdBVDV) expressing the BVDV antigens, whereas calves in the positive control group received a commercial BVDV MLV vaccine. Calves in the negative control group were inoculated with the recombinant AdLuc. The calves were boosted with the respective priming inoculum and dose.

### Cellular and humoral immune responses

Two weeks post-priming and bi-weekly thereafter, PBMCs were isolated to evaluate and quantify proliferation of BVDV-specific T-cell responses as previously described [58]. The PBMCs (2.5 x 10^5^ cells/well) were cultured for 72 hr. at 37°C in triplicate wells of round-bottom 96-well plates in a total volume of 100 μl of complete RPMI-1640 (cRPMI) medium containing 2.5 μg/ml defined BVDV CD4^+^ T-cell epitope peptides [32]. The positive control was cRPMI medium containing 1.3 μg/ml concanavalin A (ConA), whereas medium alone served as a negative control. Cells were labeled with 0.25 μCi of ^3^H-thymidine for 6 hr., harvested using a semi-automatic cell harvester (Tomtec Life Sciences, Hamden, CT), and the incorporated ^3^H-thymidine was counted with a Micro-Beta liquid scintillation counter (Perkin Elmer, Waltham, MA). The incorporation of ^3^H-thymidine by the proliferating PBMCs was presented as mean counts per minute (cpm) of triplicate wells (±1 SD).

The PBMCs were also used to quantify BVDV-specific IFN-γ-secreting cells by EliSpot assay as previously described [58]. The PBMCs (2.5 x 10^5^ cells/well) were seeded into triplicate wells of MultiScreen-HA plates (EMD Millipore, Billerica, MA) in a final volume of 100 μl cRPMI medium containing 2.5 μg/ml BVDV CD4^+^ T-cell epitope peptides. The positive control was 1.3 μg/ml ConA, whereas medium alone served as a negative control. The plates were incubated for 36 hr. at 37°C, washed, developed, and dried overnight as previously described [58]. Following quantification of the spots using an EliSpot reader (AID, Diagnostika GmbH, Germany), the mean number of spots in the negative control wells was subtracted from the mean number of spots in the cognate test wells to determine the mean number of BVDV-specific IFN-γ-secreting PBMCs and the results were presented as the mean number of spot-forming cells/10^6^ PBMCs.

Sera from blood collected two weeks post-boost and one week pre-challenge were tested to determine BVDV-1 and BVDV-2 neutralizing antibody titers using BVDV-1 (Singer, NADL, BJ, TGAC, CA0401186a) and BVDV-2 (890, 1373, A125) strains as previously described [61, 62]. Briefly, serum was heat inactivated at 56°C for 30 min, and 25 μl of each serum was serially diluted (2-fold) in cell culture media without FBS in 96-well microtiter plates. Stock BVDV virus containing 100 TCID_50_/25μl was added to each test well. In each test, a positive control serum was also included. This serum/virus mixture was incubated for 1 hr., at 37°C, MDBK cells added, and the plates were incubated at 37°C in a humidified atmosphere of 5% CO_2_ for 72 hr. The cells were observed daily for CPE for cytopathic strains, whereas the non-cytopathic strains were detected by Immuno-peroxidase assay [63]. The results were presented as serum neutralization titers (SNT) [64].

### Clinical parameters: Viremia/WBC-platelet counts

Calves were observed daily pre-immunization, post-immunization and post-challenge for coughing, nasal discharge and diarrhea. Rectal temperature post-challenge was taken daily [65]. To determine virus titers post-challenge, blood was collected in vacutainer tubes (containing Sodium-EDTA) by jugular venipuncture, freeze-thawed to lyse cells, centrifuged and supernatants were used to determine BVDV titers as previously described [66]. Whole blood was used to determine CBC using Cell-Dyn 3700 analyzer (Abbott Diagnostics, Lake Forest, IL 60045, USA) with veterinary package as bovines for automated counts (WBC, RBC, Hgb, MCV, PLT). Thin blood smears were stained with Giemsa for differential white blood cell counts [67]. Platelet count verification, WBC count verification, RBC and WBC morphology was evaluated microscopically.

### Persistence of recombinant adenovirus in cattle

Presence of recombinant replication-incompetent adenovirus in cattle was tracked for three weeks post-inoculation by rescue of virus from tissue biopsies taken from the intradermal inoculation site. Briefly, recombinant adenovirus (5 x 10^9^ ifu) was inoculated (I.D) into nine marked sites on the neck of four steers. One skin biopsy was taken from each site using a 4mm Biopsy Punch (American Screening, Shreveport, LA) on days 1–7, 14, and 21. In addition, skin biopsies were concurrently collected from the flank region of each steer to serve as negative controls. The steers were euthanized three weeks post-inoculation and samples of draining lymph node and spleen were collected. The fresh tissue samples collected were snap frozen in liquid nitrogen, ground and then resuspended in 1 ml DMEM (Invitrogen). Following centrifugation, supernatants were filtered through 0.22 μm pore membrane, and 0.5 ml was added to one well of HEK-293A cells (which supports adenovirus replication) grown in 12-well plates. Supernatant from HEK-293A cells infected overnight with the recombinant adenovirus, and subjected to the same treatment as above was used as a positive control. Three days post-infection, presence of adenovirus in the HEK-293A cells was evaluated by immunocytometric analysis using the rabbit anti-adenovirus polyclonal IgGs as above. Medium from the above HEK-293A cells was used to infect fresh cells, and seven days later the above process was repeated to confirm presence or absence of adenovirus.

### Statistical analysis

Analysis of Variance (ANOVA) followed by Tukey’s multiple comparison test was used to analyze the significance of the differences in BVDV-specific immune responses and disease indices between the treatments (groups A and B) and the negative control (group C) using GraphPad Prism 6 (Version 6.07, GraphPad Software, Inc. La Jolla, USA). Statistical significance was considered when P < 0.05.

### Ethics statement

The study was conducted in accordance with the Public Health Service Policy on Humane Care and Use of Laboratory Animals as specified in the Health Research and Extension Act of 1985 (Public Law 99–158) or in accordance with the U.S Department of Agriculture policies as required by the Animal Welfare Act of 1966 (7.USC.2131 *et seq*) as amended in 1970, 1976, and 1985. The research protocol: AUP21010-65 was reviewed and approved by the Texas A&M University Institutional Animal Care and Use Committee to ensure compliance with PHS standards. All animal care facilities are inspected twice per year. The facilities and procedures for maintenance and care of animals are accredited by the American Association for Accreditation of Laboratory Animal Care. Efforts were made to minimize suffering, and at the completion of the study, the calves were euthanized with an overdose of sodium pentobarbital. This method is approved by the Panel on Euthanasia of the American Veterinary Medical Association.

## Results and Discussion

### Expression constructs encoding novel mosaic BVDV antigens

**Fig 1:**
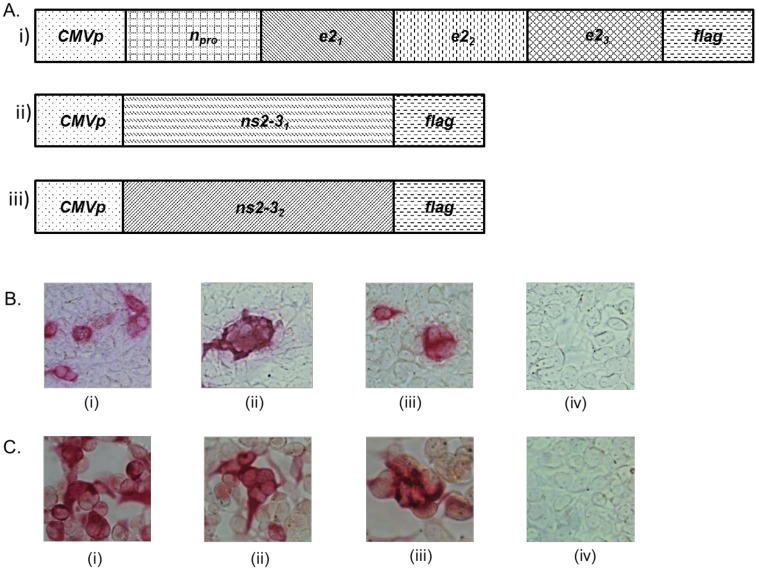
BVDV antigen expression constructs. A) Schematic diagram of expression cassettes encoding BVDV mosaic antigens: i) Composition of the *n*_*pro*_*e2*_*1-3*_, chimeric gene: *n*_*pro*_ encodes N^pro^ antigen; *e2*_*1*_ encodes a mosaic BVDV-1a E2 envelope glycoprotein; *e2*_*2*_ encodes a mosaic BVDV-1b E2 envelope glycoprotein; whereas *e2*_*3*_ encodes a mosaic BVDV-2 E2 envelope glycoprotein. ii) *ns2-3*_*1*_ encodes a mosaic BVDV-1 nonstructural protein 2–3. iii) *ns2-3*_*3*_ encodes a mosaic BVDV-2 nonstructural protein 2–3. A gene (*flag*) encoding the FLAG tag was fused in-frame at the 3’ end of each chimeric gene for tracking protein expression and transcription was under the direction of the CMV promoter (*CMVp*). The genes were cloned into adenovirus backbone plasmid vector and the resultant constructs were designated pAdN^pro^E2^1-3^, pAdNS^2-31^, and pAdNS^2-32^, respectively. B) Protein expression by recombinant plasmid constructs: The plasmid DNA constructs encoding the three genes described in (A) above were transfected into HEK-293A cell monolayers and protein expression was evaluated by immunocytometric analysis using anti-FLAG M2-AP Conjugate as follows: HEK-293A cells monolayers were transfected with the following constructs: i) pAdN^pro^E2^1-3^; ii) pAdNS^2-31^; iii) pAdNS^2-32^; and iv) pAd vector (negative control). C) Protein expression by recombinant adenovirus constructs: HEK-293A cells monolayers were infected with the following recombinant adenovirus: i) AdN^pro^E2^1-3^; ii) AdNS^2-31^; iii) AdNS^2-32^; and iv) Ad-Luciferase. Protein expression was evaluated by immunocytometric analysis as above.

Three synthetic genes (designated *n*_*pro*_*e2*_*1-3*_, *ns2-3*_*1*,_ and *ns2-3*_*2*_) encoding novel BVDV mosaic antigens were designed as depicted in Fig 1A. The *n*_*pro*_*e2*_*1-3*_ chimeric gene encodes the N-terminal protease fragment (N^pro^), a consensus BVDV-1a envelope glycoprotein E2 mosaic gene (*e2*_*1*_), a consensus BVDV-1b envelope glycoprotein E2 mosaic gene (*e2*_*2*_), and a consensus BVDV-2 envelope glycoprotein E2 mosaic gene (*e2*_*3*_) fused in-frame to *flag*-tag. The *ns2-3*_*1*_ DNA fragment encodes a consensus BVDV-1 Nonstructural protein 2–3 fused in-frame to *flag*-tag, whereas the *ns2-3*_*2*_ DNA fragment encodes a consensus BVDV-2 Nonstructural protein 2–3 fused in-frame to *flag*-tag (Fig 1A).

### Expression of the mosaic BVDV antigens

Immunocytometric analysis of HEK-293A cells transfected with the pAdN^pro^E2^1-3^, pAdNS^2-31^, or pAdNS^2-32^ constructs probed with anti-FLAG mAb confirmed that each construct expressed the encoded antigen (Fig 1B). Similarly, immunocytometric analysis of HEK-293A cells infected with the AdN^pro^E2^1-3^, AdNS^2-31^, or AdNS^2-32^ recombinant adenoviruses probed with anti-FLAG mAb confirmed protein expression (Fig 1C). Analysis of replication competency confirmed that the AdN^pro^E2^1-3^, AdNS^2-31^, and AdNS^2-32^ recombinant adenoviruses were replication-incompetent.

### Novel mosaic BVDV antigens are recognized by multiple BVDV-specific antibodies

**Fig 2:**
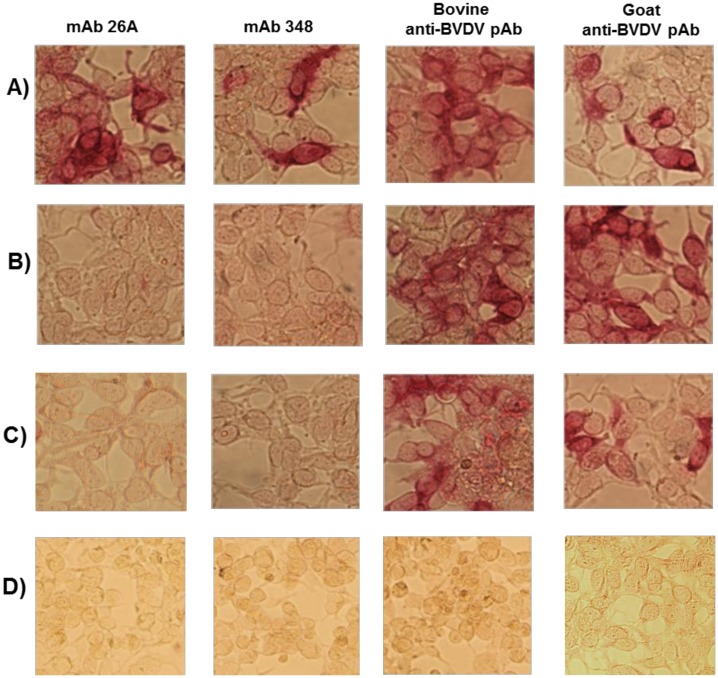
Validation of B-cell epitopes in the mosaic BVDV antigens. Authenticity of the adenovirus-expressed novel BVDV mosaic antigens was confirmed by immunocytometric analysis using E2-specific neutralizing monoclonal antibodies 26A and 348 (both neutralize BVDV-1 & 2); bovine anti-BVDV hyper-immune serum (generated by immunizing steers multiple times with BVDV-1 & 2 vaccines followed by boosting with killed diverse BVDV-1 & 2 strains and then challenged with wild type BVDV-1 & 2 strains (The sera have high BVDV-1 & 2 neutralizing titers [59]); and goat anti-BVDV polyclonal serum generated against multiple wild-type BVDV-1 & 2 strains. A) HEK-293A cells expressing N^pro^E2^1-3^; B) HEK-293A cells expressing NS^2-31^; C) HEK-293A cells expressing NS^2-32^; and D) HEK-293A cells expressing luciferase.

Authenticity of the mosaic antigens (N^pro^E2^1-3^, NS^2-31^, and NS^2-32^) expressed by the recombinant adenoviruses was confirmed by immunocytometric analysis of infected HEK-293A cells probed with BVDV neutralizing monoclonal antibodies and polyclonal sera raised against diverse BVDV strains (Fig 2). Anti-BVDV polyclonal sera from immunized goat and cattle reacted with all three recombinant antigens (N^pro^E2^1-3^, NS^2-31^, and NS^2-32^), whereas monoclonal antibodies 26A and 348, specific for the glycoprotein E2, reacted with N^pro^E2^1-3^ antigen only (Fig 2A). The outcome confirmed that neutralization epitopes in the mosaic N^pro^E2^1-3^ antigen were correctly expressed, and that the NS^2-31^ and NS^2-32^ mosaic antigens were specifically recognized by anti-BVDV polyclonal sera. Thus, these antigens were expected to induce authentic BVDV-specific immune responses in cattle. This expected outcome was consistent with previous demonstration that multicomponent mosaic antigens generated using this strategy elicit broadly protective pathogen-specific immune responses [68–70].

### Novel mosaic BVDV antigens are recognized by BVDV-specific T lymphocytes

**Fig 3:**
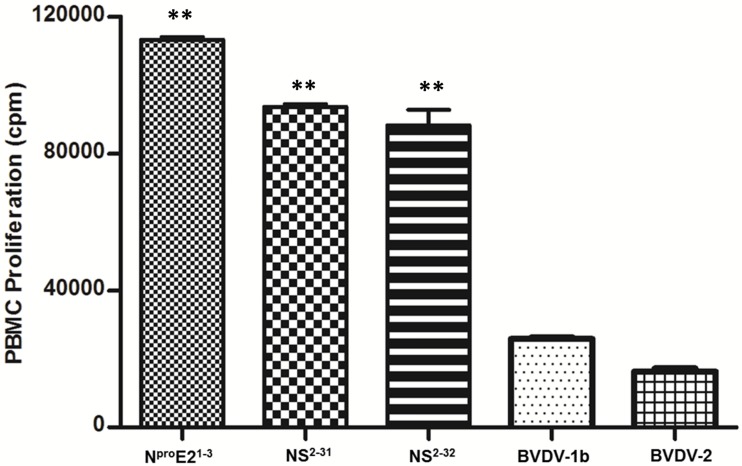
Validation of mosaic antigens using BVDV-specific T-cells. Authenticity of T-cell epitopes in the mosaic BVDV antigens was validated by proliferation assay using PBMCs from a BVDV-1 & 2 hyper-immune steer [59]. The data shown is minus background counts from negative control (media alone) treatment. The asterisks denote a statistically significant difference (P<0.01) between the proliferation induced by the N^pro^E2^1-3^, NS^2-31^ and the NS^2-32^ antigens and both whole killed viruses BVDV-1b and BVDV-2. This outcome is representative of assays conducted using PBMCs from other BVDV immune steers.

The N^pro^E2^1-3^, NS^2-31^, and NS^2-32^ antigens stimulated robust proliferation of PBMCs from BVDV-immunized steers (Fig 3). The recall responses stimulated by the mosaic antigens were significantly (P<0.01) higher than the responses elicited by whole killed BVDV-1b or BVDV-2, suggesting that the mosaic antigens are likely to prime and amplify robust antigen-specific immune responses *in vivo* (Fig 3). These outcomes showed that the mosaic antigens were properly processed to generate peptides that were presented by MHC molecules to cognate BVDV-specific memory T-cells. Previous studies have shown that mosaic antigens are processed by host APCs to generate relevant peptides for MHC presentation to elicit protective T-cell responses [71, 72].

### Mosaic antigens elicited stronger BVDV-specific T-cell immune responses

**Fig 4:**
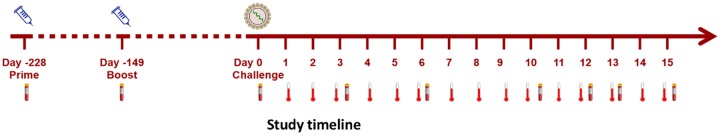
Immunization timeline. On day -228 pre-challenge, cattle in the treatment group were vaccinated with a cocktail of the recombinant adenoviruses expressing mosaic BVDV antigens (AdBVDV), whereas positive control cattle received a commercial MLV BVDV vaccine. Negative control cattle were inoculated with the recombinant AdLuc. On day -149 pre-challenge, the cattle were boosted with the respective priming inoculum and dose (Table 1). On day 0, all the cattle were challenged by intranasal delivery of a BVDV-1373 using an atomizer. Blood samples were collected on selected days (0, 3, 6, 10, 12, 13 and 15), whereas clinical observations and rectal temperatures were monitored and recorded daily from days 1–15 post-challenge.

**Fig 5:**
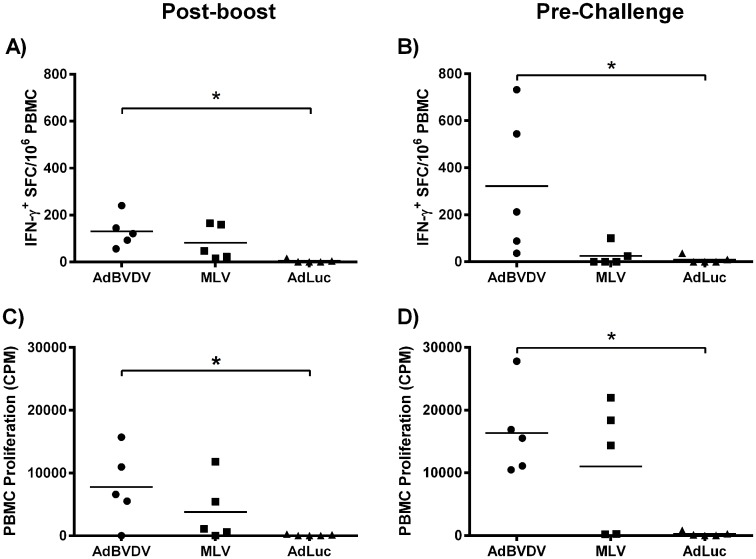
Mosaic BVDV vaccine elicited strong T-cell responses. Immunization of cattle with adenovirus-vectored mosaic BVDV vaccine primed and expanded BVDV-specific T-cells. A commercial MLV BVDV vaccine served as a positive control, whereas AdLuciferase served as a negative control. EliSpot assays were used to evaluate BVDV-specific IFN-γ-secreting PBMC responses against defined BVDV T-cell epitopes and data is shown for A) One-week post-boost; and B) Day 0 of challenge. Outcome is presented as IFN-γ^+^ SFC/10^6^ PBMC. Cell proliferation assays were used to evaluate BVDV-specific PBMC responses and data is shown for C) One-week post-boost; and D) one-week pre-challenge. Proliferation of the PBMCs in response to defined BVDV T-cell epitopes is presented as the means ± standard deviations of ^3^H-thymidine incorporation by the cells from triplicate wells. In both assays, medium alone served as the negative control and the data shown is minus media background counts. The group mean is represented by a bar. Asterisks denote statistically significant differences, *P<0.05.

Immunogenicity and protective efficacy of the AdN^pro^E2^1-3^, AdNS^2-31^, and AdNS^2-32^ recombinant adenovirus cocktail, designated AdBVDV, was evaluated in steers using a homologous prime-boost immunization regimen (Table 1 and Fig 4). One week after the AdBVDV vaccinees were boosted, the cocktail elicited higher, but not significantly different, BVDV-specific IFN-γ-secreting PBMCs as well as BVDV-specific PBMC proliferation compared to the vaccinees that received the commercial MLV BVDV vaccine (Fig 5A & 5C). The mean responses mounted by the AdBVDV vaccinees, but not the MLV vaccinees, were significantly higher (P<0.05) than the negative controls. Before challenge (five months after the AdBVDV vaccinees were boosted) the AdBVDV-induced mean IFN-γ^+^ response had increased and was significant (P<0.05) compared to the negative controls, whereas the mean IFN-γ^+^ response in the MLV vaccinees had already declined (Fig 5B). This decrease in the mean IFN-γ response in the MLV BVDV vaccine treatment group one week before challenge, might have had an impact on clearance of the challenge virus.

The mean BVDV-specific PBMC proliferation increased in both the vaccinated groups, but only the AdBVDV- and not the MLV-induced response was significantly different (P<0.05) from the AdLuc control group (Fig 5D). The increase in mean IFN-γ response and PBMC proliferation in the AdBVDV treatment group at five months post-boost, were not significantly different from the responses recorded at one week post-boost (Fig 5).

### Mosaic antigens elicited cross-protective BVDV-specific antibody responses

**Fig 6:**
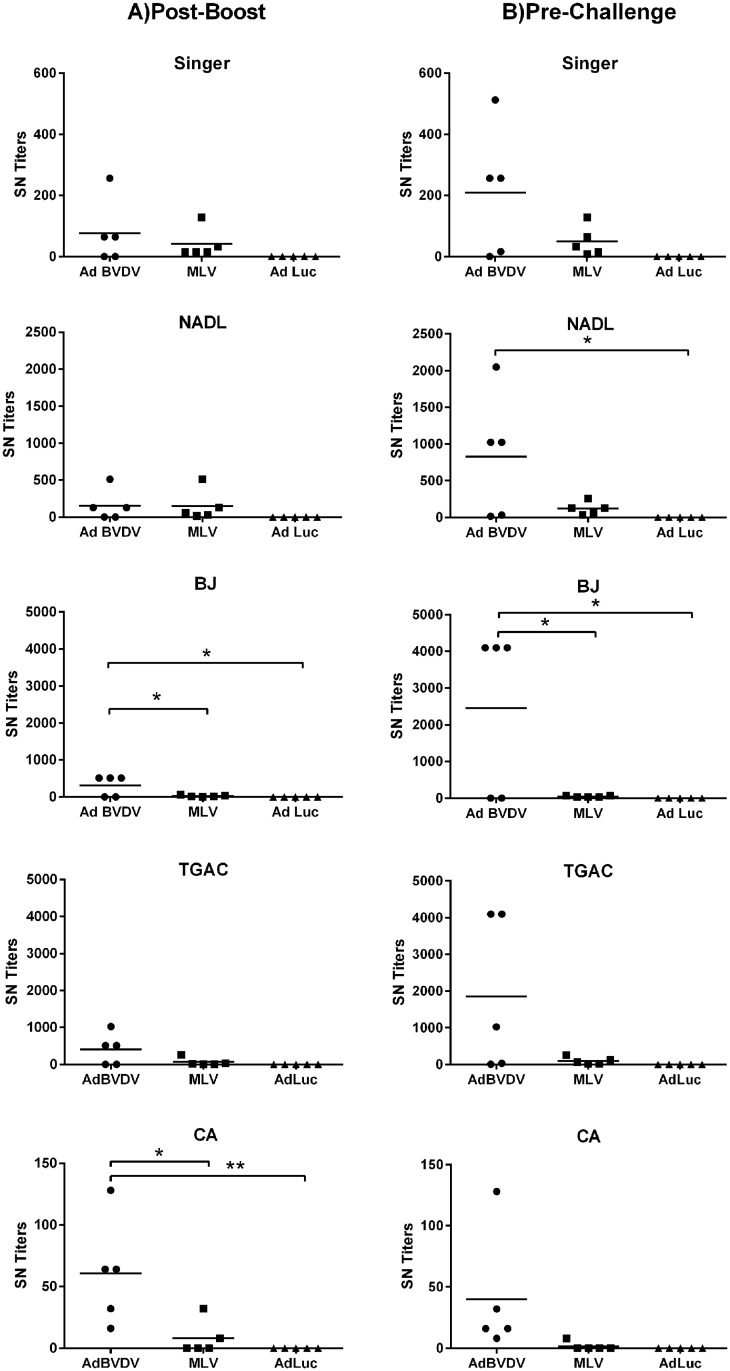
Mosaic BVDV vaccine induced BVDV-1 specific neutralizing antibodies. Serum neutralization assays were used to evaluate BVDV-1-specific neutralization titers at A) One-week post-boost; and B) one-week pre-challenge against five BVDV type 1 strains. Mean group titers are represented by the bars. Statistically significant differences between the groups are denoted by asterisks. *P<0.05; **P<0.01.

**Fig 7:**
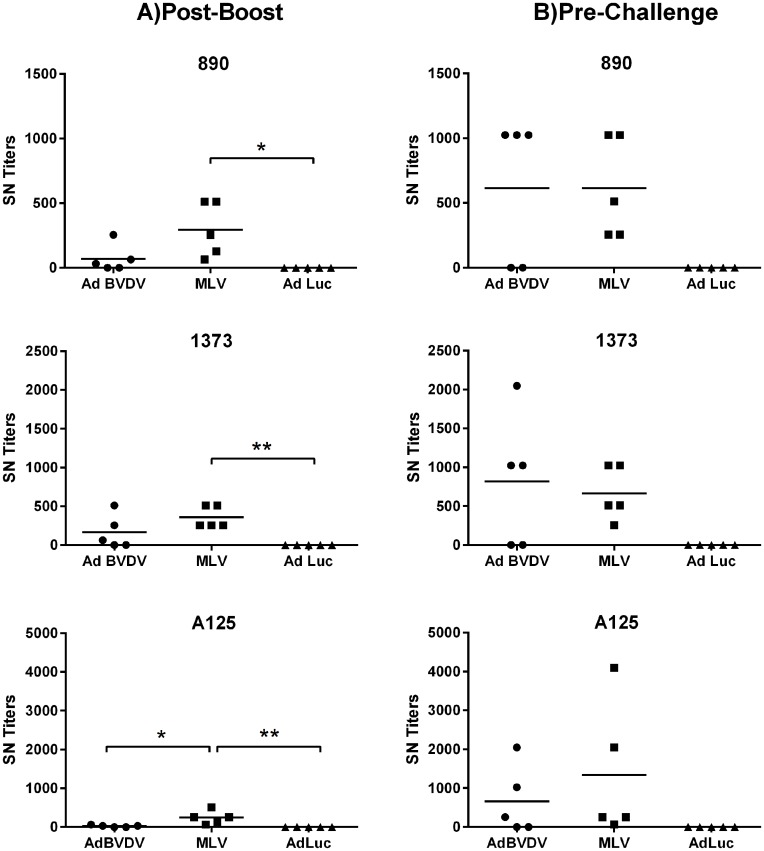
Mosaic BVDV vaccine induced BVDV-2 specific neutralizing antibodies. BVDV-2-specific neutralization titers against three BVDV type 2 strains were evaluated at A) two weeks post-boost; and B) one week pre-challenge. Mean group titers are represented by the bars. Statistically significant differences between the groups are denoted by asterisks *P<0.05; **P<0.01.

Following boosting of the AdBVDV vaccinees, the levels of BVDV neutralizing serum antibodies against five BVDV-1 strains and three BVDV-2 strains were evaluated at one-week post-boost and one-week pre-challenge (Figs 6 and 7). The adenovirus cocktail induced higher mean neutralizing antibody titers post-boost against all BVDV-1 strains compared to the responses stimulated by the commercial MLV BVDV vaccine and the AdLuc controls. The difference between the mean titers however, was significant only for the non-cytopathic BVDV-1b BJ (P<0.05) and BVDV CA0401186a strains (AdBVDV vs MLV, P<0.05; AdBVDV vs AdLuc, P<0.01) (Fig 6A). Furthermore, the mean AdBVDV titers increased up to five months post-boost (one-week pre-challenge) against 4 of 5 BVDV-1 strains whereas, the mean MLV titers either remained the same or declined. These mean AdBVDV titers remained significantly higher (P<0.05) than the MLV vaccinees and the AdLuc controls for the BJ strain, and only the AdLuc controls for the cytopathic BVDV-1a NADL strain. Interestingly, for all three BVDV-2 strains, the mean titers of the MLV vaccinees were higher (in contrast to BVDV-1) than the AdBVDV vaccinees post-boost. These mean MLV titers were significantly higher (P<0.05) than the AdBVDV vaccinees only for strain A125 and significantly higher than the AdLuc controls for all three strains (P<0.05 for strain 890; P<0.01 for strains 1373 and A125). The mean BVDV-2-specific titers in both the AdBVDV vaccinees as well as the MLV vaccinees increased before challenge. Thus overall, the AdBVDV vaccine cocktail was able to induce high titers against all 8 BVDV strains tested in 3 out of 5 calves, whereas the MLV vaccine was able to induce high titers against only BVDV-2 strains. It is also noteworthy that the 3 AdBVDV vaccinees had substantially higher neutralizing titers (1:1024–1:2048) when compared to the MLV vaccinees (1:32–1:256) against the NADL strain which is a component of the commercial MLV vaccine they received.

### Clinical observations, hematology and viremia

**Fig 8:**
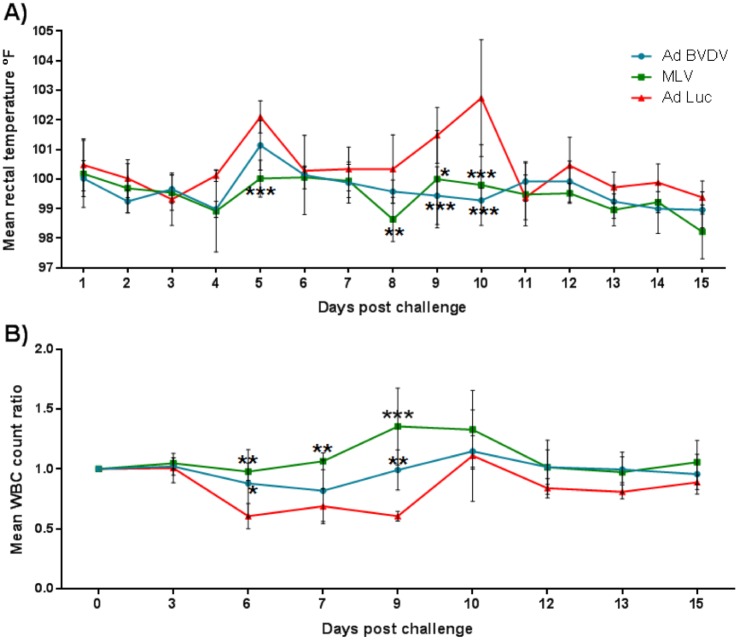
Clinical manifestations post-challenge. A) Mean rectal temperature fluctuation; and B) Mean change ratios of white blood cell counts in the vaccinated and negative control groups post-challenge. Asterisks denote statistically significant differences as compared to the negative controls. *P<0.05; **P<0.01 and ***P<0.001.

Following the BVDV challenge, there were no obvious differences in clinical score among all the animals, however, characteristic biphasic pyrexia was observed for the negative controls but not for the AdBVDV or the MLV vaccinees (Fig 8A). On day 5 post-challenge, the transient rise in mean rectal temperatures of the negative controls was significantly higher (P<0.001) than the MLV vaccinees but not the AdBVDV vaccinees. The mean rectal temperatures for the negative controls rose again on day 9, peaked at day 10 and normalized by day 11 post-challenge. The mean temperatures of the controls were significantly higher than AdBVDV vaccinees on days 9 (P<0.05) and 10 (P<0.001) post challenge, and the MLV vaccinees on days 8 (P<0.01), 9 (P<0.001) and 10 (P<0.001) post-challenge (Fig 8A). The negative control animals also exhibited transient leucopenia from days 6 to 9 post-challenge with a 32–40% reduction against baseline (day 0) white blood cell (WBC) counts. This reduction of WBCs in the negative controls was significant compared to the AdBVDV vaccinees on days 6 (P<0.05) and 9 (P<0.01) post-challenge, and the MLV vaccinees on days 6 (P<0.01), 7 (P<0.01) and 9 (P<0.001) post-challenge (Fig 8B). There was no significant difference in platelet counts among the treatment groups post-challenge. On days 7 and 10 post-challenge, no virus was detected in all the AdBVDV vaccinees (Table 2). However, BVD virus was detected from the blood of one of the steers that received the commercial MLV BVDV vaccine on day 7 but not on day 10 post-challenge, and from the blood of all the negative controls up to day 15 post-challenge (Table 2).

**Table 2:** Virus isolation from calves on day 7 and day 10 post-challenge.

Cattle ID	Vaccine	Viremia	
		Day 7 post-challenge	Day 10 post-challenge
4	AdBVDV	-	-
12	AdBVDV	-	-
13	AdBVDV	-	-
22	AdBVDV	-	-
23	AdBVDV	-	-
3	BVDV MLV	-	-
14	BVDV MLV	-	-
19	BVDV MLV	-	-
24	BVDV MLV	-	-
27	BVDV MLV	+ (10^−2^)	-
10	AdLuciferase	+ (10^−3^)	+ (10^−3^)
18	AdLuciferase	+ (10^−3^)	+ (10^−3^)
25	AdLuciferase	+ (10^−3^)	+ (10^−3^)
28	AdLuciferase	+ (10^−3^)	+ (10^−3^)
29	AdLuciferase	+ (10^−3^)	+ (10^−3^)

Viremia in blood samples taken on days 7 and 10 post-challenge was evaluated by immunocytometric analysis of MBDK cells probed with goat anti-BVDV polyclonal serum. The dilution at which the samples were positive is specified. Sample dilutions further than 10^−3^ were not tested.

### Recombinant adenovirus inoculated intradermally is short lived

**Fig 9:**
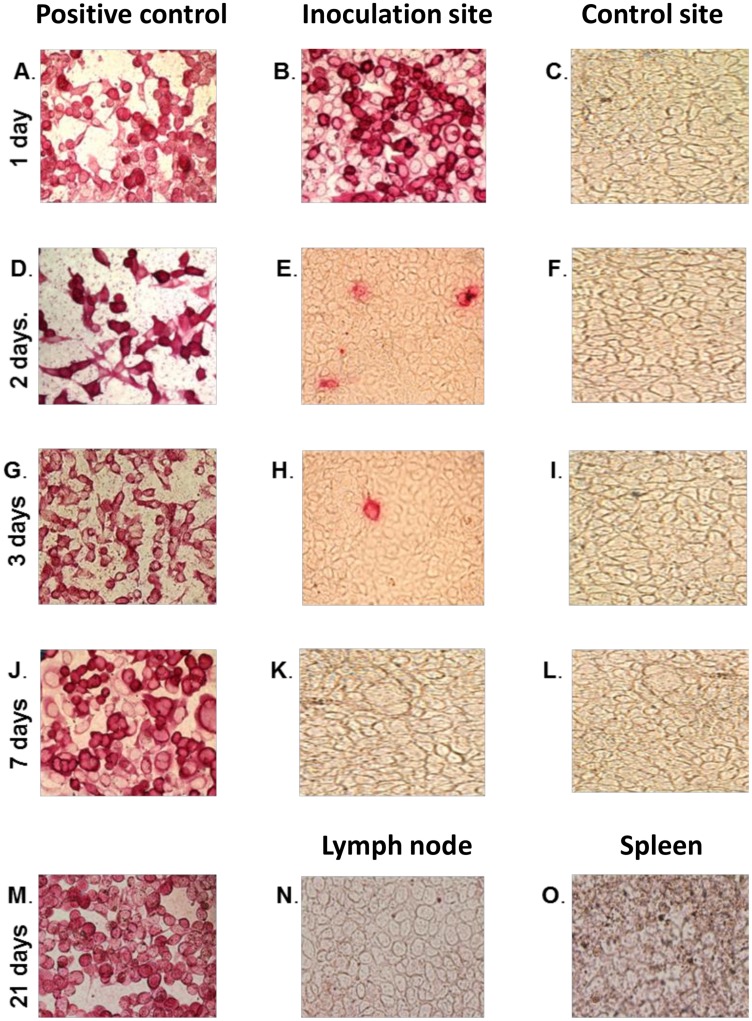
Persistence of replication-incompetent adenovirus in cattle. Viable recombinant adenovirus inoculated intradermally is only recoverable within three days. Presence of adenovirus rescued from tissue samples of four steers at defined time points was tracked by immunocytometric analysis of HEK-293A cells. Representative data from one steer is shown: A, D, G, J, and M are positive controls at 24 hr., 48 hr., 72 hr., day 7, and day 21, respectively. B, E, H, and K, are skin biopsies taken from the inoculation sites on the neck of the steers at 24 hr., 48 hr., 72 hr., and day 7, respectively, whereas C, F, I, and L, are cognate control skin biopsies taken concurrently from the flank. N and O are draining lymph node and spleen samples, respectively, collected three weeks post-inoculation.

Persistence of recombinant replication-incompetent adenovirus at the intradermal inoculation site, the draining lymph node, and the spleen was monitored by HEK-293A cell-dependent virus rescue followed by immunocytometric analysis using adenovirus-specific polyclonal antibody. One-day post-inoculation, adenovirus was readily recovered from the skin biopsies collected from the inoculation sites, but not from the control sites (Fig 9B and 9C). Virus recovery decreased drastically by day two post-inoculation and very few viral particles were recovered at day three (Fig 9E and 9H). No virus was recoverable from all skin biopsies collected on days 4–7 post-inoculation (Fig 9K). Skin biopsies collected on days 14 and 21, and draining lymph node and spleen samples collected on day 21 were all negative (Fig 9N and 9O). The medium from the HEK-293A cells used to test the samples collected on days 4–7, 14, and 21, was negative after a second round of screening. These outcomes are consistent with previous findings in rodents [73]. Given that ABSL2 biocontainment is required for *in vivo* studies using the replication-incompetent adenovirus, data from this pilot study suggest that it is safe to downgrade biocontainment after seven days post-inoculation. However, the fate of the vector genome in cattle and environmental risk assessment will need to be determined.

## Conclusions

The purpose of this study was to develop an efficacious prototype BVDV vaccine which a) overcomes the several disadvantages associated with the MLV vaccine mentioned previously and b) provides broad protection against multiple BVDV genotypes. To this end, we designed mosaic polypeptide consensus sequences of highly immunogenic BVDV antigens such as N^pro^, E2 glycoprotein and the Nonstructural protein 2–3 based on multiple genotypes. We selected live replication deficient adenovirus as a vector for delivery of these antigens to prime strong humoral as well as cell mediated immune responses. Polyclonal anti-BVDV sera and monoclonal anti-E2 antibodies strongly recognized these mosaic antigens by immunocytometric analysis. Furthermore, PBMCs from BVDV immune steers proliferated strongly upon stimulation by these mosaic antigens. The above outcomes confirmed the authenticity of both B-cell and T-cell epitopes in all the mosaic antigens.

Calves immunized with a cocktail of recombinant adenoviruses expressing these antigens had stronger IFN-γ^+^ and proliferation responses to defined BVDV CD4^+^ T-cell epitopes as compared to calves vaccinated with the commercial BVDV MLV vaccine. In addition, the AdBVDV vaccinees had higher serum neutralizing titers against BVDV-1 than the MLV vaccinees. In case of BVDV-2, the MLV vaccinees had higher mean titers one week post-boost, but the AdBVDV mean titers increased over time and before challenge were equivalent or higher than the MLV vaccinees for 2 of 3 strains tested. Importantly, both BVDV-1 and BVDV-2 neutralizing antibody titers along with the cellular IFN-γ^+^ and proliferation immune responses considerably increased for up to five months post-boost (one week before challenge) in most AdBVDV vaccinees, whereas only the BVDV-2 specific titers and the mean proliferation responses amplified in the MLV vaccinees. Upon challenge with a BVDV-2a strain, both vaccinated groups showed no clinical signs of infection. The negative controls however, had a mild fever on day 5 post-challenge followed by a more severe pyrexia on day 10 post-challenge. Moreover, the negative controls also had significantly lower WBC counts than both vaccinated groups. Rapid clearance of virus is an attractive trait in a BVDV vaccine. All the AdBVDV vaccinees had cleared the virus as early as 7 days post-challenge, whereas one MLV vaccinee was still viremic on day 7 but not on day 10 post-challenge. All negative controls remained viremic up to day 15 post-challenge. With regards to the safety concern and ABSL2 biocontainment when using human Ad5 as a delivery vector, we showed that the replication-incompetent Ad5 virus is cleared from the inoculation site within four days post-injection and is not recovered from either the draining lymph node or the spleen after 21 days post-inoculation.

Overall, data from this study showed that the AdBVDV prototype vaccine is more immunogenic and offers better cross-protection than the commercial MLV vaccine in terms of cell mediated and neutralizing antibody responses. As far as protective efficacy is concerned, the AdBVDV vaccine performed at par if not better than the MLV vaccine upon challenge by BVDV-2a strain. Notably, this study is the first to report heterologous protection using subunit BVDV vaccines. Future studies with larger animal sample sizes, different vaccine doses and challenge with diverse BVDV strains need to be conducted to further optimize the AdBVDV prototype vaccine.

The protective potential of the BVDV E2 antigen has been successfully demonstrated in the past using various delivery platforms like live-vectors, DNA immunizations or as a recombinant protein produced in different expression systems [74–77]. Current efforts are now focused on enhancing this potential using modern adjuvants and antigen carriers such as PRR activators, APC targeting molecules and silica nanoparticles [78–81]. This study highlights the cross-protective potential of the novel mosaic polypeptides and is the first to report heterologous protection using subunit BVDV vaccines. Thus, future studies using these mosaic polypeptide sequences in conjunction with modern immune-response enhancing strategies may lead to a very effective and cross-protective BVDV vaccine.
